# Use of Unamplified RNA/cDNA–Hybrid Nanopore Sequencing for Rapid Detection and Characterization of RNA Viruses

**DOI:** 10.3201/eid2208.160270

**Published:** 2016-08

**Authors:** Andy Kilianski, Pierce A. Roth, Alvin T. Liem, Jessica M. Hill, Kristen L. Willis, Rebecca D. Rossmaier, Andrew V. Marinich, Michele N. Maughan, Mark A. Karavis, Jens H. Kuhn, Anna N. Honko, C. Nicole Rosenzweig

**Affiliations:** US Army Edgewood Chemical Biological Center, Aberdeen Proving Ground, Maryland, USA (A. Kilianski, P.A. Roth, A.T. Liem, J.M. Hill, K.L. Willis, R.D. Rossmaier, A.V. Marinich, M.N. Maughan, M.A. Karavis, C.N. Rosenzweig);; Defense Threat Reduction Agency, Fort Belvoir, Virginia, USA (K.L. Willis);; National Institutes of Health, Fort Detrick, Frederick, Maryland, USA (J.H. Kuhn, A.N. Honko)

**Keywords:** Ebola virus, viruses, nanopore sequencing, Venezuelan equine encephalitis virus, RNA virus, genomics, sequencing, fieldable platform, Zika virus

## Abstract

Nanopore sequencing, a novel genomics technology, has potential applications for routine biosurveillance, clinical diagnosis, and outbreak investigation of virus infections. Using rapid sequencing of unamplified RNA/cDNA hybrids, we identified Venezuelan equine encephalitis virus and Ebola virus in 3 hours from sample receipt to data acquisition, demonstrating a fieldable technique for RNA virus characterization.

Portable and reliable molecular epidemiology techniques and field approaches for assessing virus genomes are desired to inform clinical diagnostics and public health operations. Need for such methods has been highlighted by the recent Middle East respiratory syndrome and Ebola virus disease (EVD) epidemics, during which it became necessary to characterize novel viruses and to evaluate genetic drift, transmission chains, and zoonotic introductions.

To determine if nanopore sequencing can be used as an accelerated viral genome sequencing tool, we utilized a rapid cDNA/RNA–hybrid library preparation procedure to sequence cell cultures of Venezuelan equine encephalitis virus vaccine (VEEV) strain TC-83 or Ebola virus (EBOV) isolate Makona-C05 stock IRF0137. To evaluate nanopore sequencing for rapid, field-deployable pathogen characterization, we collected raw read data and statistics for VEEV and EBOV sequence runs on the MinION sequencing device (Oxford Nanopore Technologies, Oxford, UK). To determine the level of identification and accuracy of genome characterization over sequencing runtime, these reads were then mapped to VEEV and EBOV genomes and to reference databases (RefSeq [www.ncbi.nlm.nih.gov/RefSeq/]). From the results of these analyses, we determined that the current and future versions of nanopore sequencing technology can be used to rapidly identify and characterize pathogens. .

## The Study

This approach for pathogen identification and characterization differs from the previously used methods on the MinION platform. Biased techniques, such as amplicon sequencing, have proven to be effective in complex sample backgrounds in which titers of the target pathogen might be low, but such approaches limit characterization to known pathogens and require additional viral genome amplification (*1*–*4*). Unbiased techniques that require viral genome amplification (*5*) or that have been optimized for bacterial genomes (*6*,*7*) require longer sample and library preparation times, but can detect low pathogen titers or create highly accurate genomic data. We sequenced unamplified poly(A)-tailed viral RNA using rapid cDNA library preparation coupled with real-time data analysis to determine its potential application for pathogen genomic characterization.

VEEV has a single-stranded, linear, poly(A)-tailed RNA genome. Thus, poly-dT primers can be used for cDNA production without further genomic RNA manipulation. The workflow to isolate the RNA and prepare it for sequencing (Technical Appendix) took ≈3 hours from the initiation of sample processing to data acquisition on MinION (Figure, panel A). The sequencing of VEEV genomic RNA/cDNA hybrids attained in hours by using MinION revealed reads that mapped to the VEEV TC-83 genome within minutes by using the LAST (Computational Biology Research Consortium, Tokyo, Japan) multiple sequence alignment program (Technical Appendix; Figure, panel B [*2*,*4*,*6*]). The coverage increased from 15–60 min from the 3′ end of the VEEV genome with reads aligning directionally from the 3′ to 5′ end of the VEEV genome (Figure, panel B). These alignment characteristics are indicative of the poly-dT priming strategy for poly(A)-tailed RNA.

**Figure:**
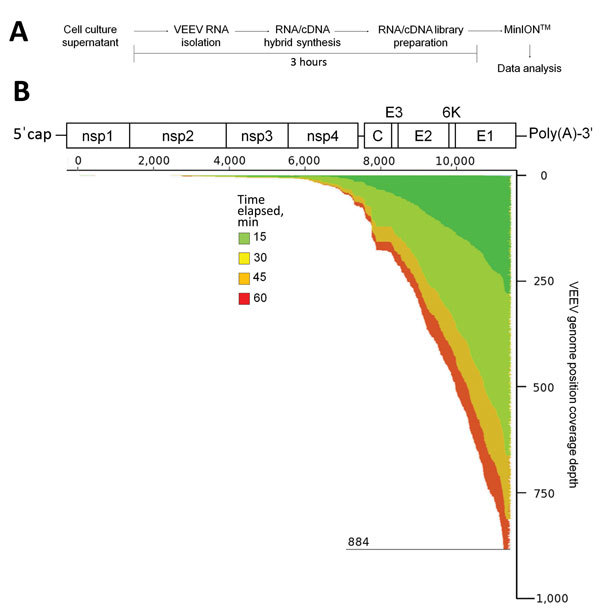
Use of unamplified RNA/cDNA−-hybrid nanopore sequencing for genomic characterization of Venezuelan equine encephalitis virus (VEEV) TC-83. A) Sample preparation workflow for nanopore sequencing. First, viral RNA from BHK21 cell cultures of VEEV TC-83 was isolated, then single strand complimentary DNA (cDNA) was synthesized. The resulting RNA/cDNA hybrids were then prepared for nanopore sequencing and sequenced with data analysis occurring in real time. B) Genome organization and sequencing coverage over time of VEEV TC-83. VEEV is an alphavirus; its genome consists of a single strand of positive-sense RNA that can be translated into a polyprotein. Translation is critically dependent on the genomic 3′ poly(A)-tail. This tail can be used for reverse transcription priming by using poly-(dT) primers that anneal to it. Read data was aligned to VEEV TC-83 (accession number L01443) by using the multiple sequence alignment program LAST (Computational Biology Research Consortium, Tokyo, Japan [Technical Appendix]). The coverage map shows the depth of genome coverage over 15, 30, 45, and 60 minutes of sequencing runtime, with the greatest depth observed at the 3′ end of the VEEV genome. Nsp, nonstructural protein; C, capsid; E, envelope

To determine if the reads generated from VEEV TC-83 would align to the correct viral genome within a set of reference sequences, we used the viral genome reference sequences (http://www.ncbi.nlm.nih.gov/genomes/GenomesGroup.cgi?taxid=10239&opt=Virus), plus the VEEV TC-83 genome database (GenBank accession no. L01443). We then used LAST to align nanopore reads against this set of references (Technical Appendix). These alignments were used to generate a top hit table and the associated read alignment statistics against each hit. VEEV TC-83 was the top hit based on LAST alignment versus virus RefSeq genome sequences; wild-type VEEV placed second (Table). VEEV TC-83 was also identified as the top hit when the 15- and 60–min sequencing datasets were compared with alphavirus genome sequences (Technical Appendix) (Table), demonstrating accuracy and depth achieved in a short time. We also analyzed the VEEV TC-83 dataset using the cloud-based metagenomic detection platforms Pathosphere (*8*) and One Codex (www.onecodex.com), and found that the sample contained VEEV (Technical Appendix).

**Table:** Alignment statistics for detection of VEEV TC-83 and EBOV/Mak-C05 using unamplified RNA/cDNA-hybrid nanopore sequencing*

Virus samples and time points, min	Top hits (GenBank accession no.)	LAST score	Total bases mapped, %	Coverage, %	Average base depth	Per read accuracy, %
VEEV TC-83 (GenBank accession no. L01443)						
Viral genomes (RefSeq databases†)						
15	VEEV TC-83 (L01443)	138,321	5.54	76.14	50.94x	59–80
	VEEV WT (NC_001449.1)	789	0.05	18.59	1.76x	60–78
60	VEEV TC-83 (L01443)	419,153	17.17	78.54	153.16x	57–80
	VEEV WT (NC_001449.1)	1,182	0.08	32.12	1.82x	58–78
Alphavirus genomes						
15	VEEV TC-83 (L01443)	31,320	1.13	48.92	16.21x	67–69
	VEEV E541/73 (AF093102.1	6,463	0.27	95.07	5.26x	62–73
	VEEV 71–180 (AF069903.1)	5,865	0.22	30.18	5.08x	65–73
60	VEEV TC-83 (L01443)	96,348	3.55	48.92	50.84x	62–74
	VEEV E541/73 (AF093102.1)	21,411	0.89	99.91	16.36x	61–73
	VEEV 71–180 (AF069903.1)	16,429	0.64	51.04	8.78x	65–73
EBOV/Mak-C05 (GenBank accession no. KX000400)						
Viral genomes (RefSeq databases)						
15	EBOV/Mak-137 (KX000400)	529	0.11	9.29	1.00x	68
	Bovine herpesvirus (NC_024303.1)	73	0.02	0.18	1.00x	67
60	EBOV/Mak-137 (KX000400)	2,371	0.53	22.23	2.09x	66–71
	Bovine herpesvirus (NC_024303.1)	239	0.04	0.27	1.58x	67–74

*VEEV, Venezuelan equine encephalitis virus; EBOV, Ebolavirus; LAST (Computational Biology Research Consortium, Tokyo, Japan), multiple sequence alignment program. †RefSeq, NCBI Reference Sequence Database (http://www.ncbi.nlm.nih.gov/RefSeq/).

Molecular epidemiology, including use of viral genomics, played a major role during the 2013–2016 EVD response, informing contact tracing, diagnostic operability, and public health measures (*9*–*11*). To determine if EBOV is amenable to the same rapid sequencing methodology that was used for VEEV, unamplified negative-stranded RNA isolated from EBOV in Trizol (Thermofisher Scientific, http://www.thermofisher.com/us/en/home/brands/product-brand/trizol.html) was poly(A)-tailed, a single complementary strand of cDNA synthesized, and RNA/cDNA hybrids sequenced. The EBOV samples sequenced on MinION rapidly provided usable, accurate data, despite less raw data than the VEEV TC-83 dataset (137kbp for EBOV versus 2.4Mbp for VEEV at 60 min). Using 15-and 60-min time points and an identical alignment strategy to VEEV TC-83 above, we detected EBOV as the top hit within the sequencing dataset when compared to all virus RefSeq sequences (Table).

Despite success against the RefSeq database, the lack of depth within the dataset did not enable differentiation between the EBOV isolate sequenced here and the >1,500 EBOV draft genomes sequenced during the 2013–2016 outbreak (*10*,*12*,*13*), which indicates a limitation in this sequencing approach for negative-stranded RNA viruses. The poly(A)-tailing method was chosen because the reverse transcription primer adapters designed by Oxford Nanopore were developed to interact directly with the motor protein necessary for guiding DNA through the nanopores. This method greatly reduced preparation time and eliminated need for adaptor ligation reagents. This approach can be revisited for sequencing negative-strand RNA viruses (*2*,*5*). Despite this limitation, the RefSeq alignments and nearest neighbor calls were possible with limited data, demonstrating the potential power of long-read rapid sequencing on nanopore platforms.

## Conclusions

The current Middle East respiratory syndrome, EVD, and Zika virus disease outbreaks illustrate the necessity for rapid characterization of pathogens for environmental detection, clinical evaluation, and epidemiologic investigation. To determine whether nanopore sequencing can fill this role in a fieldable platform, we tested an RNA/cDNA–hybrid sequencing approach on VEEV TC-83 (a positive-stranded RNA virus) and EBOV (a negative-stranded RNA virus) prepared from cell-culture supernatants. This method definitively identified VEEV TC-83 and differentiated it from wild-type VEEV in ≈3 hours, including only 15 min of data acquisition on MinION. VEEV TC-83 was also differentiated from other alphavirus genomes, facilitating strain-level identification of TC-83. EBOV was also identified rapidly by this approach, differentiating the virus in the sample analyzed here from available virus reference genomes. However, variant/isolate level characterization was not possible due to limited data generated from the RNA/cDNA–hybrid approach.

The method applied here is greatly accelerated compared to traditional next-generation sequencing library preparation, and was used with reagents and equipment suitable for austere conditions (e.g., little need for cold chain, steps not requiring PCR). This study confirmed the possibility of accurate RNA virus genome characterization from RNA/cDNA hybrids by using limited sample manipulation, albeit from relatively pure samples. If samples derived directly from clinical matrices (e.g., blood, saliva) were used, this method would probably not support the necessary depth to characterize virus genomes unless the pathogen titer within these samples was high. As the depth of sequence data obtained from nanopore sequencing approaches continues to improve (*14*) and other pore types (such as RNA-specific sequencing pores) are integrated into commercial products, these unamplified techniques can transition from the laboratory to the field for more complex analysis.

Utilization of nanopore sequencing in Western Africa (*2*,*3*) has demonstrated potential for its use, and newly developed methods like this RNA/cDNA–hybrid approach can be integrated into fieldable protocols. For the emerging Zika virus, insufficiently high virus titers in clinical samples usually necessitates virus culture before genomic sequencing (*15*). Genomic Zika virus isolate characterization efforts would greatly benefit from the approaches outlined here, especially regarding materials needed for genomic library preparation and the time reduction for strain-level identification (*15*). By preparing and sequencing RNA/cDNA hybrids, the sample-to-answer time for RNA sequencing is greatly reduced, providing pathogen identification and characterization rapidly to inform future decision making.

Technical Appendix Description of determining the ability of using nanopore sequencing to provide rapid genomic data for RNA virus pathogens, supported by data collection and analysis techniques.
