# Supplementary material for: Use of Unamplified RNA/cDNA–Hybrid Nanopore Sequencing for Rapid Detection and Characterization of RNA Viruses
Source: Emerg Infect Dis. 2016 Aug;22(8):1448–51. doi: 10.3201/eid2208.160270 (PMC4982148; doi:10.3201/eid2208.160270)
Supplement: Technical Appendix — Description of determining the ability of using nanopore sequencing to provide rapid genomic data for RNA virus pathogens, supported by data collection and analysis techniques. [file 16-0270-Techapp-s1.pdf]

DOI: http://dx.doi.org/10.3201/eid2208.160270

Use of Unamplified RNA/cDNA-Hybrid Nanopore Sequencing for Rapid Detection and Characterization of RNA Viruses

Technical Appendix

Viral Growth and RNA Isolation

To determine the ability of nanopore sequencing to provide rapid genomic data on RNA virus pathogens, a workflow was adopted and developed from cDNA sequencing protocols created by Oxford Nanopore Technologies (Oxford, UK) (MAP SEQ-002) (Figure 1, panel A). In brief, Venezuelan equine encephalitis virus (VEEV) vaccine strain TC-83 and Ebola virus (EBOV) variant Makona isolate C05 stock IRF0137 (EBOV/Mak-C05) were grown and RNA isolated from clarified cell-culture supernatants. VEEV TC-83 was prepared from stocks derived from United States Army Venezuelan equine encephalitis virus TC-83 stocks. One MOI of VEEV TC-83 was adsorbed on Vero E6 monolayers for 2 hours. After 48 h of incubation in Minimum Essential Medium-α + 10% fetal bovine serum (GIBCO, Gaithersburg, MD; ThermoFisher Scientific, Pittsburgh, PA), cell culture supernatants were collected and clarified by centrifugation at 650 × *g* for 10 min at 4°C. RNA was isolated from cell-culture supernatant using the QIAamp MinElute virus spin kit (QIAGEN) for isolated VEEV particles.

The C05 isolate of the Makona variant of Ebola virus (full designation: Ebola virus/H.sapiens-tc/GIN/2014/Makona-C05, abbreviation: EBOV/Mak-C05) was isolated in 2014 in Vero E6 cells and kindly provided by Dr. Gary P. Kobinger (Public Health Agency of Canada, Winnipeg, Canada, BioSample: SAMN03611815, internal reference IRF0135). Vero E6 cells were used to propagate EBOV by two additional tissue culture passages in Vero E6 cells using Minimum Essential Medium-α, GlutaMAX, no nucleosides (GIBCO, ThermoFisher Scientific) supplemented with 2% US-origin, certified, heat-inactivated fetal bovine serum (HI-FBS, GIBCO, ThermoFisher Scientific). Following harvest, HI-FBS was QS'd to 10% final concentration before cryopreservation. GenBank accession no. KX000400 BioSample:

SAMN04490241, internal reference IRF0137. RNA was isolated from virus preps in 1:4 supernatant: TRIzol after RNA extraction with cleanup using RNeasy MinElute cleanup kit (QIAGEN).

RNA/cDNA-Hybrid Sequencing Preparations

A total of 250 ng of VEEV or EBOV RNA were either directly added to a single-strand cDNA reaction (VEEV) primed using poly-dT primers provided by Oxford Nanopore Technologies (ONT; DEV-MAP003) or poly(A)-tailed (*Escherichia coli* poly(A) polymerase, (New England BioLabs) before addition (EBOV). cDNA synthesis was performed using SuperScript II reverse transcription 18064–014 (Life Technologies, Carlsbad, CA) per standard manufacturers protocols at 50°C for 50 min, followed by 70°C for 15 min. After cDNA synthesis, cDNA/RNA hybrids were prepared for nanopore sequencing (DEV-MAP003) by purifying RNA/cDNA hybrids using 0.7× Agencourt AmPure XP beads (Beckman Coulter, Fullerton, CA) followed by 2× 80% ethanol washes. Purified RNA/cDNA hybrids were then incubated with binding buffer for 45 min (ONT; DEV-MAP003), motor protein for 5 min (ONT; DEV-MAP003), then loading buffer for 5 min (ONT; DEV-MAP003) per the manufacturers protocol (ONT; DEV-MAP003). Prepared libraries were then diluted per manufacturers protocol with water and fuel mix (ONT; DEV-MAP003) to a final volume of 300 μl, with 150 μL sequencing solution loaded with p1000 tips onto individual MinION flow cells (7.3) for sequencing.

Data Collection and Analysis

Data were collected in real time using Oxford Nanopore software (VEEV: MinKNOW: 0.50.1.15, Metrichor: 1.13.1; EBOV: MinKNOW: 0.48.2.14, Metrichor: 1.10.1) and analyzed for genome alignment using LAST-648 (*1*) (lastal) with the options (-s 2 -T 0 -Q 0 -a 1). Duplications in the alignments were removed using last-map-probs (*1*). These methods are identical to ones used for previous work with amplicon and native nucleic acid sequencing (*2*–*6*). Nanopore reads were aligned against the Viral Genomes database (*7*) of viral reference genome sequences (data for complete genomes: Viruses (taxid 10239); 6,635 total entires, with the 2 stock genomes added from this study) and top hits and alignment statistics were generated.

VEEV reads were also aligned against a database of alphavirus genome sequences (1,440 total entries; detailed below). The read files, associated data and scripts are available at GenBank EU accession numbers SAMEA3865262 (VEEV) and SAMEA3865263 (EBOV) for nanopore data and NCBI BioProject PRJNA311755 and GenBank accession no. KX000400 for the EBOV reference. Sample use of Pathosphere (*8*) for analysis is publically available at www.pathosphere.org under the manuscript header. One Codex analysis (*9*) is also publically available at https://app.onecodex.com/analysis/public/57c08743784b41d3. All reference files used for the analysis above are available as 'viralRefSeq.fa' and 'all_alphavirus_complete.fasta' attached here. Scripts are available below in addition to being hosted at www.pathosphere.org.

References

1. Kiełbasa SM, Wan R, Sato K, Horton P, Frith MC. Adaptive seeds tame genomic sequence comparison. Genome Res. 2011;21(3):487–93. http://dx.doi.org/10.1101/gr.113985.110 **PMID: 21209072**

2. Hoenen T, Groseth A, Rosenke K, Fischer RJ, Hoenen A, Judson SD, et al. Nanopore sequencing as a rapidly deployable Ebola outbreak tool. Emerg Infect Dis. 2016;22; Epub ahead of print.

3. Laver TW, Caswell RC, Moore KA, Poschmann J, Johnson MB, Owens MM, et al. Pitfalls of haplotype phasing from amplicon-based long-read sequencing. Sci Rep. 2016;6:21746. http://dx.doi.org/10.1038/srep21746 **PMID: 26883533**

4. Laver T, Harrison J, O'Neill PA, Moore K, Farbos A, Paszkiewicz K, et al. Assessing the performance of the Oxford Nanopore Technologies MinION. Biomol Detect Quantif. 2015;3:1–8. http://www.ncbi.nlm.nih.gov/pmc/articles/PMC4691839/ **PMID: 26753127**

5. Wang J, Moore NE, Deng Y-M, Eccles DA, Hall RJ. MinION nanopore sequencing of an influenza genome. Front Microbiol. 2015;6:766. http://dx.doi.org/10.3389/fmicb.2015.00766 **PMID: 26347715**

6. Quick J, Ashton P, Calus S, Chatt C, Gossain S, Hawker J, et al. Rapid draft sequencing and real-time nanopore sequencing in a hospital outbreak of Salmonella. Genome Biol. 2015;16:114. http://www.ncbi.nlm.nih.gov/pubmed/26025440 **PMID: 26025440**

7. Brister JR, Ako-adjei D, Bao Y, Blinkova O. NCBI viral genomes resource. Nucleic Acids Res. 2014;43:D571–7. http://www.ncbi.nlm.nih.gov/pubmed/25428358 http://dx.doi.org/10.1093/nar/gku1207

8. Kilianski A, Carcel P, Yao S, Roth P, Schulte J, Donarum GB, et al. Pathosphere.org: pathogen detection and characterization through a web-based, open source informatics platform. BMC Bioinformatics. 2015;16:416. http://dx.doi.org/10.1186/s12859-015-0840-5 **PMID: 26714571**

9. Minot SS, Krumm N, Greenfield NB. One Codex: A sensitive and accurate data platform for genomic microbial identification. bioRxiv. 2015 Sep 28; Epub ahead of print. http://biorxiv.org/content/early/2015/09/28/027607

Scripts

```
#!/bin/bash # last_time_alignment_pipeline
# # This is the main script that aligns reads
using LAST according to timestamps #
produced by the ExtractTimesFromReads
program. It converts the fast5 files to a #
fastq, extracts only the reads that were
produced before specified time argument,
# prepares the reference database for last,
aligns and then outputs the top 10 reference
hits from
# the database.
#
# Dependencies that must be installed:
LAST, JAVA, perl, bash, HDF5 libraries.
#
# This program was developed exclusively
with government funds by
# OptiMetrics, Inc. in support of U.S. Army
Edgewood Chemical Biological
# Center.
#
# Copyright (C) 2016 OptiMetrics, Inc.
#
# This program is free software: you can
redistribute it and/or modify
# it under the terms of the GNU General
Public License as published by
# the Free Software Foundation, version 3 of
the License.
#
# This program is distributed in the hope
that it will be useful,
# but WITHOUT ANY WARRANTY;
without even the implied warranty of
# MERCHANTABILITY or FITNESS FOR
A PARTICULAR PURPOSE. See the
# GNU General Public License for more
details.
#
# You should have received a copy of the
GNU General Public License
# along with this program. If not, see
<http://www.gnu.org/licenses/>.
#
set -o nounset;
set -o errexit;
args=($@);
len=${#args[@]};
min_scripts=”$(cd “$(dirname
“${BASH_SOURCE[0]}”)” && pwd)”;
if [ $len -lt 4 ] || [ $len -gt 4 ]
then
echo “Usage:
last_time_alignment_pipeline.sh <fast5
```

```
input directory> <reference fasta> <time in seconds> <output directory>"
exit;
fi
fast5_dir=${args[0]};
reference=${args[1]};
time=${args[2]};
output_dir=${args[3]};
mkdir -p $output_dir;
cd $output_dir;
reads=$output_dir/reads.fq;
if [ ! -e $reads ]
then
echo "converting fast5 to fastq ....";
ls $fast5_dir/*.fast5 | xargs -I [] java -jar -Djava.library.path=$min_scripts/lib $min_scripts/Fast5toFastq.jar [] > $output_dir/reads.fq
fi
timestamps=$output_dir/timestamps.csv;
if [ ! -e $timestamps ]
then
echo "extracting timestamps from fast5 ....";
ls $fast5_dir/*.fast5 | xargs -I [] java -Djava.library.path=$min_scripts/lib -cp $min_scripts/lib/:$min_scripts/source extracttimesfromreads.ExtractTimesFromReads [] > $timestamps;
fi
echo "creating last db reference ...";
lastdb ref $reference;
echo "filtering reads file by time ...";
perl $min_scripts/filter_reads_by_time.pl $time $time $timestamps $reads $output_dir/reads.$time.fna
echo "running last alignment ...";
lastal -r1 -a1 -b1 -q1 -Q0 ref $output_dir/reads.$time.fna > $output_dir/align.$time.maf
echo "running last-map-probs";
/common/bin/last-658/last-map-probs $output_dir/align.$time.maf > $output_dir/align.$time.nodups.maf
echo "getting top scores ...";
perl $min_scripts/findTopAlignmentScores.pl $output_dir/align.$time.nodups.maf $output_dir/topscores.$time.txt
echo "DONE!";
#$Log$
/**
* Fast5toFastq
*
* This program extracts the fastq data from a fast5 file that has been
* processed using the Metrichor analysis from Oxford Nanopore.
*
* This program was developed exclusively with government funds by
* OptiMetrics, Inc. in support of U.S. Army Edgewood Chemical Biological
* Center.
*
* Copyright (C) 2016 OptiMetrics, Inc.
*
* This program is free software: you can redistribute it and/or modify
* it under the terms of the GNU General Public License as published by
* the Free Software Foundation, version 3 of the License.
* This program is distributed in the hope that it will be useful,
* but WITHOUT ANY WARRANTY; without even the implied warranty of
* MERCHANTABILITY or FITNESS FOR A PARTICULAR PURPOSE. See the
* GNU General Public License for more details.
*
* You should have received a copy of the GNU General Public License
* along with this program. If not, see <http://www.gnu.org/licenses/>.
*
*/
package fast5tofastq;
import java.io.BufferedReader;
import java.io.File;
import java.io.FileReader;
import java.util.StringTokenizer;
```

```
import ncsa.hdf.object.h5.*; // include the
HDF5 object package
import ncsa.hdf.hdf5lib.*; // include the Java
HDF5 interface
*
*/
public class Fast5toFastq
{
public static void main(String[] argv)
{
if (argv.length != 1)
{
System.out.println("Usage: Fast5toFastq
<fast5_filename>");
System.out.println("Output prints to
STDOUT");
try
{
}
catch (Exception e)
{
}
return;
}
// create an H5File object
H5File h5file = new H5File(argv[0],
HDF5Constants.H5F_ACC_RDONLY);
try
{
BufferedReader reader = null;
String path =
Fast5toFastq.class.getProtectionDomain().ge
tCodeSource().getLocation().toURI().getPat
h();
File configFile = new
File(path.substring(0,path.lastIndexOf("/")+
1) + "fast5tofastq.conf");
try
{
reader = new BufferedReader(new
FileReader(configFile));
}
catch (Exception e)
{
System.out.println("Config file
fast5tofastq.conf must be in the same
location as the fast5tofastq jar file.");
return;
}
h5file.open();
while (reader.ready())
{
String fastqPath = reader.readLine();
// open file and retrieve the file structure
try
{
H5ScalarDS obj = (H5ScalarDS)
h5file.get(fastqPath);
String [] s = (String [])obj.read();
for(int i = 0; i < s.length; i++)
System.out.println(s[i]);
}
catch (Exception e)
{
// This path doesn't exist in the file. Do
nothing, and move on.
}
}
reader.close();
}
catch (Exception ex)
{
System.err.println(ex);
}
try { h5file.close(); }
catch (Exception ex) {}
}
}
/**
* ExtractTimesFromReads
*
* This program extracts the read names and
read times from a fast5 file
* that has been processed using the
Metrichor analysis from Oxford Nanopore.
*
* This program was developed exclusively
with government funds by
* OptiMetrics, Inc. in support of U.S. Army
Edgewood Chemical Biological
```

```
* Center.
*
* Copyright (C) 2016 OptiMetrics, Inc.
*
* This program is free software: you can
redistribute it and/or modify
* it under the terms of the GNU General
Public License as published by
* the Free Software Foundation, version 3 of
the License.
* This program is distributed in the hope
that it will be useful,
* but WITHOUT ANY WARRANTY;
without even the implied warranty of
* MERCHANTABILITY or FITNESS FOR
A PARTICULAR PURPOSE. See the
* GNU General Public License for more
details.
*
* You should have received a copy of the
GNU General Public License
* along with this program. If not, see
<http://www.gnu.org/licenses/>.
*
*/
package extracttimesfromreads;
import java.io.BufferedReader;
import java.io.File;
import java.io.FileReader;
import java.util.List;
import ncsa.hdf.object.h5.*; // include the
HDF5 object package
import ncsa.hdf.hdf5lib.*; // include the Java
HDF5 interface
import ncsa.hdf.object.Attribute;
public class ExtractTimesFromReads
{
public static void main(String[] argv)
{
if (argv.length != 1)
{
System.out.println("Usage:
ExtractTimesFromReads
<fast5_filename>");
System.out.println("Output prints to
STDOUT");
System.exit(0);
}
// create an H5File object
H5File h5file = new H5File(argv[0],
HDF5Constants.H5F_ACC_RDONLY);
String name="";
try
{
BufferedReader reader = null;
String path =
ExtractTimesFromReads.class.getProtection
Domain().getCodeSource().getLocation().to
URI().getPath();
File configFile = new
File(path.substring(0,path.lastIndexOf("/")+
1) + "fast5tofastq.conf");
try
{
reader = new BufferedReader(new
FileReader(configFile));
}
catch (Exception e)
{
System.out.println("Config file
fast5tofastq.conf must be in the same
location as the fast5tofastq jar file.");
return;
}
h5file.open();
while (reader.ready())
{
String fastqPath = reader.readLine();
// open file and retrieve the file structure
try
{
H5ScalarDS obj = (H5ScalarDS)
h5file.get(fastqPath);
String [] s = (String [])obj.read();
name = s[0].substring(0, s[0].indexOf('\n'));
}
catch (Exception e)
{
// This path doesn't exist in the file. Do
nothing, and move on.
}
}
```

```
reader.close();
H5Group obj = null;
for(int i = 0; i < 1000; i++)
{
String readString = “Read_”+ i;
//System.err.println(readString);
obj = (H5Group)
h5file.get(“Analyses/EventDetection_000/R
eads/”+readString);
if(obj !=null)
{
break;
}
}
if(obj == null)
{
//System.err.println(name);
throw new Exception(“Read does not
exist.”);
}
List<Attribute> list = obj.getMetadata();
long startTime= –1;
long duration= –1;
for(int i = 0; i < list.size(); i++)
{
if (list.get(i).toString().equals(“start_time”))
{
startTime =
((long[])list.get(i).getValue())[0];
//System.err.println(“start_time” + ” = ” +
startTime);
}
if (list.get(i).toString().equals(“duration”))
{
duration = ((long[])list.get(i).getValue())[0];
//System.err.println(“duration” + ” = ” +
duration);
}
}
obj = (H5Group)
h5file.get(“UniqueGlobalKey/channel_id”);
list = obj.getMetadata();
double samplingRate = –1;
for(int i = 0; i < list.size(); i++)
{
if
(list.get(i).toString().equals(“sampling_rate”
))
{
samplingRate =
((double[])list.get(i).getValue())[0];
//System.err.println(“sampling_rate” + ” = ”
+ samplingRate);
}
}
double time= ((double)(startTime +
duration))/samplingRate;
System.out.println(name + “,”+time);
}
catch (Exception ex)
{
System.err.println(name);
System.err.println(ex);
ex.printStackTrace();
}
try { h5file.close(); }
catch (Exception ex) {}
}
}
#!/usr/bin/perl
# Filter_reads_by_time
#
# This program will pull out reads that were
generated before the specified time
# by the specified range and output it to a
fasta file.
#
# This program was developed exclusively
with government funds by
# OptiMetrics, Inc. in support of U.S. Army
Edgewood Chemical Biological
# Center.
#
# Copyright (C) 2016 OptiMetrics, Inc.
#
# This program is free software: you can
redistribute it and/or modify
# it under the terms of the GNU General
Public License as published by
# the Free Software Foundation, version 3 of
the License.
```

```
#
# This program is distributed in the hope
that it will be useful,
# but WITHOUT ANY WARRANTY;
without even the implied warranty of
# MERCHANTABILITY or FITNESS FOR
A PARTICULAR PURPOSE. See the
# GNU General Public License for more
details.
#
# You should have received a copy of the
GNU General Public License
# along with this program. If not, see
<http://www.gnu.org/licenses/>.
#
eval \'exec /usr/bin/perl -S $0 “$@”'
if 0; # not running under some shell
use strict;
use warnings;
my $len=scalar(@ARGV);
if ($len < 5 || $len > 6){
&printUsage();
}
my $time=$ARGV[0];
my $range=$ARGV[1];
my $timestamps_csv=$ARGV[2];
my $reads_input=$ARGV[3];
my $reads_output=$ARGV[4];
sub printUsage {
print STDOUT “Usage:
filter_sam_by_time.pl <time> <range>
<timestamps_csv> <sam_input>
<sam_output>\n”; exit(–1);
}
my %tstamps;
### Program runs here ###
open(TIME,”<$timestamps_csv”);
while(<TIME>){
my $ln=$_;
chomp $ln;
if($ln=~m/^([^\,]+)\,(.*)$/){
my $sid=$1;
my $tstamp=$2;
$sid=~s/\_strand//g;
if($tstamp<$time && $tstamp>=($time-
$range)){
$tstamps{$sid}=$tstamp;
}
}
} close TIME;
open(READSIN,”<$reads_input”);
open(READSOUT,”>$reads_output”);
while(<READSIN>){
my $ln=$_;
chomp $ln;
my $readName=$ln;
if(defined($tstamps{$readName})){
$ln=~s/^\@/\>/;
print READSOUT $ln.”\n”;
$ln=<READSIN>;
chomp $ln;
print READSOUT $ln.”\n”;
my $null=<READSIN>;
$null=<READSIN>;
}
}
close READSIN;
close READSOUT;
#!/usr/bin/perl
# findTopAlignmentScores
#
# This program pools the scores of each last
alignment by reference.
# It then picks the top 10 references based
on their scores.
#
# This program was developed exclusively
with government funds by
# OptiMetrics, Inc. in support of U.S. Army
Edgewood Chemical Biological
# Center.
#
# Copyright (C) 2016 OptiMetrics, Inc.
#
# This program is free software: you can
redistribute it and/or modify
# it under the terms of the GNU General
Public License as published by
# the Free Software Foundation, version 3 of
the License.
#
```

```
# This program is distributed in the hope
that it will be useful,
# but WITHOUT ANY WARRANTY;
without even the implied warranty of
# MERCHANTABILITY or FITNESS FOR
A PARTICULAR PURPOSE. See the
# GNU General Public License for more
details.
#
# You should have received a copy of the
GNU General Public License
# along with this program. If not, see
<http://www.gnu.org/licenses/>.
#
eval \'exec /usr/bin/perl -S $0 "$@"'
if 0; # not running under some shell
use strict;
use warnings;
my $len=scalar(@ARGV);
if ($len < 2 || $len > 2){
&printUsage();
}
my $alignment=$ARGV[0];
my $output=$ARGV[1];
sub printUsage {
print STDOUT "Usage:
findTopAlignmentScores.pl <alignment>
<output>\n"; exit(–1);
}
open(FIN,"<$alignment");
my $last_score=0;
my %scores;
my $got_score=0;
while(<FIN>){
my $line=$_;
chomp $line;
if($line=~m/^a score\=(\d+)/){
$last_score=$1;
$got_score=1;
}
elsif($line=~m/^s\ ([^\ ]+\)/ &&
$got_score){
$got_score=0;
my $gi=$1;
if(!defined($scores{$gi})){
$scores{$gi}=$last_score;
}else{
$scores{$gi}+=$last_score;
}
}
}
close FIN;
open(FOUT,">$output");
my @keys=
sort{$scores{$b}<=>$scores{$a}}
keys(%scores);
my @vals=@scores{@keys};
for(my $i=0; $i<10; $i++){
if(defined($keys[$i])){
print FOUT $keys[$i]." score\
".$vals[$i]."\n";
}
}
close FOUT;
```
